# In-depth quantification of bimanual coordination using the Kinarm exoskeleton robot in children with unilateral cerebral palsy

**DOI:** 10.1186/s12984-023-01278-6

**Published:** 2023-11-11

**Authors:** Lisa Decraene, Jean-Jacques Orban de Xivry, Lize Kleeren, Monica Crotti, Geert Verheyden, Els Ortibus, Hilde Feys, Lisa Mailleux, Katrijn Klingels

**Affiliations:** 1 Department of Rehabilitation Sciences, Research Group for Neurorehabilitation, KU Leuven, 3000 Leuven, Belgium; 2 REVAL-Rehabilitation Research Centre, Faculty of Rehabilitation Sciences, Hasselt University, 3590 Diepenbeek, Belgium; 3 Child and Youth Institute, KU Leuven, 3000 Leuven, Belgium; 4 Department of Movement Sciences, Research Group of Motor Control and Neuroplasticity, KU Leuven, 3000 Leuven, Belgium; 5 Leuven Brain Institute, KU Leuven, 3000 Leuven, Belgium; 6 Department of Development and Regeneration, KU Leuven, 3000 Leuven, Belgium; 7 Department of Pediatric Neurology, University Hospitals Leuven, 3000 Leuven, Belgium

**Keywords:** Unilateral cerebral palsy, Robotics, Bimanual coordination, Bimanual coupling, Interlimb differences

## Abstract

**Background:**

Robots have been proposed as tools to measure bimanual coordination in children with unilateral cerebral palsy (uCP). However, previous research only examined one task and clinical interpretation remains challenging due to the large amount of generated data. This cross-sectional study aims to examine bimanual coordination by using multiple bimanual robotics tasks in children with uCP, and their relation to task execution and unimanual performance.

**Methods:**

The Kinarm exoskeleton robot was used in 50 children with uCP (mean age: 11 years 11 months ± 2 years 10 months, Manual Ability Classification system (MACS-levels: l = 27, ll = 16, lll = 7)) and 50 individually matched typically developing children (TDC). All participants performed three tasks: object-hit (hit falling balls), ball-on-bar (balance a ball on a bar while moving to a target) and circuit task (move a cursor along a circuit by making horizontal and vertical motions with their right and left hand, respectively). Bimanual parameters provided information about bimanual coupling and interlimb differences. Differences between groups and MACS-levels were investigated using ANCOVA with age as covariate (α < 0.05, ${\upeta }_{p}^{2}$). Correlation analysis (r) linked bimanual coordination to task execution and unimanual parameters.

**Results:**

Children with uCP exhibited worse bimanual coordination compared to TDC in all tasks (p ≤ 0.05, ${\eta }_{p}^{2}$ = 0.05–0.34). The ball-on-bar task displayed high effect size differences between groups in both bimanual coupling and interlimb differences (p < 0.001, ${\eta }_{p}^{2}$ = 0.18–0.36), while the object-hit task exhibited variations in interlimb differences (p < 0.001, ${\eta }_{p}^{2}$ = 0.22–0.34) and the circuit task in bimanual coupling (p < 0.001, ${\eta }_{p}^{2}$ = 0.31). Mainly the performance of the ball-on-bar task (p < 0.05, ${\eta }_{p}^{2}$ = 0.18–0.51) was modulated by MACS-levels, showing that children with MACS-level lll had worse bimanual coordination compared to children with MACS-level l and/or II. Ball-on-bar outcomes were highly related to task execution (r = − 0.75–0.70), whereas more interlimb differences of the object-hit task were moderately associated with a worse performance of the non-dominant hand (r = − 0.69–(− 0.53)).

**Conclusion:**

This study gained first insight in important robotic tasks and outcome measures to quantify bimanual coordination deficits in children with uCP. The ball-on-bar task showed the most discriminative ability for both bimanual coupling and interlimb differences, while the object-hit and circuit tasks are unique to interlimb differences and bimanual coupling, respectively.

**Supplementary Information:**

The online version contains supplementary material available at 10.1186/s12984-023-01278-6.

## Background

Bimanual coordination is defined as “a unique example of collaboration between two interconnected yet functionally specialized hemispheres to accomplish goal-directed behavior by means of integration of the left and right limb movements into a functional control entity” [1]*.* It is crucial for performing daily bimanual tasks like buttoning a shirt and grooming. Two distinct types of bimanual coordination patterns exist, namely preferred and non-preferred. While preferred coordination involves simultaneously (in-phase) or alternating (anti-phase) rhythmic activation of the homologous muscles, non-preferred refers to coordination modalities that are commonly used in daily life and require practice to be performed effectively [1]. Tasks that follow a non-preferred coordination pattern consist of two different categories: bilateral symmetrical tasks where similar demands are imposed on each hand (e.g. lifting a box of the floor) and bilateral complementary tasks where demands on each hand differ (e.g. tying shoe laces) [2]. The task performance of typically developing children (TDC) evolves from a slow and sequential performance with each hand separately to a well-coordinated strategy in adulthood, due to experience and increasing prevalence of bilateral complementary tasks in daily life [3]. However, children with neurodevelopmental disorders, such as cerebral palsy (CP), may exhibit impairments in bimanual coordination, possibly causing problems in both bilateral symmetrical and complementary tasks.

CP is the most common motor disorder in children caused by a brain lesion of the developing fetal or infant brain [4]. It has a prevalence of 3 per 1000 live births [4] of which 44% has unilateral CP (uCP) [5]. Due to sensorimotor deficits on one side of the body, which are mostly present in the upper limb, children with uCP have difficulties with performing daily life activities. From the age of nine years, the spontaneous use of the non-dominant hand in bimanual activities even tends to decline, despite improvements in unimanual capacity [6]. Bimanual performance has been defined as the spontaneous use of the non-dominant hand during a bimanual activity [7]. However, in literature, bimanual performance has most commonly been assessed with the clinical Assisting Hand Assessment (AHA) [7], which has been referred to in literature as not being able to capture the precise coordination between both hands [8, 9]. To address this issue, the Two Arm Coordination Test, which requires participants to manipulate two handles simultaneously to control a pointer in a star shape, was developed [9]. Despite providing information about speed and accuracy, the outcome parameters offer limited insights into bimanual coordination [9]. Some studies investigated bimanual coordination quantitatively with a 3D motion registration during different symmetrical and asymmetrical tasks [10], showing that children with uCP have less bimanual coupling compared to TDC and thus worse coordination [11]. Bimanual coupling refers to the active spatiotemporal connection of both arms in order for them to operate as a single functional synergy [12]. Nevertheless, bimanual coupling is only one component of bimanual coordination. Interlimb differences, which refer to the differences in physical abilities between both hands during a bimanual task, can also have an important role in bimanual coordination. Although natural differences between hands exist, the reduced capacity of the non-dominant hand in children with uCP could influence the efficiency and success of bimanual coordination tasks as it has been shown in adults with stroke [12]. Interlimb differences could affect both symmetrical and complementary tasks in children with uCP. Larger differences may create synchronization problems in symmetrical tasks, while compensatory movements with the dominant hand can create larger interlimb differences in complementary tasks. As previous research in children with uCP focused mainly on bimanual coupling [11, 13], more research is needed to map both bimanual coupling and interlimb differences to fully understand the bimanual coordination abilities in this population.

It has been recently demonstrated that robotic assessments have the ability to measure bimanual coordination deficits in children with uCP [14]. Such cutting-edge technology has a number of benefits over clinical assessments, including the ability for objective and accurate assessment, which can provide detailed information about complex movements [15]. In paediatric populations, four different robotic systems have been used to assess upper limb function [16]. Nevertheless, only the Kinarm robots are able to assess both arms at the same time, hence study bimanual coordination, including both bimanual coupling and interlimb differences [16]. Despite this, the Kinarm exoskeleton robot has primarily been utilized in children with uCP to assess somatosensory and unimanual reaching deficits [17–19]. Additionally, the majority of studies only included one standardized task and analyzed a subset of the outcome parameters without providing information regarding parameter selection. Although the Kinarm exoskeleton is a valuable tool to identify bimanual coordination deficits in children with uCP, the infinite possibilities of tasks and outcome parameters on this robotic device can be a drawback. Hence, it is important to first conduct an exploratory study involving all outcome parameters, which will allow for a more substantiated selection of tasks and parameters in future studies.

In addition to exploring bimanual coordination abilities in children with uCP, it is crucial to understand the influence of these abilities on their manual ability, or their capacity to perform daily activities using both arms [20]. While previous studies have shown that children with a lower manual ability take longer to complete bimanual tasks [13], the relationship between bimanual coordination and manual ability remains unravelled. Therefore, we want to examine whether bimanual coordination is related with manual abilities as evaluated by the Manual Ability Classification System (MACS).

Next to this, it has been shown that the capacity of the non-dominant hand affects bimanual coordination in children with uCP [11]. Additionally, it has been demonstrated that children with uCP predominantly rely on their dominant hand during the performance of bimanual tasks [21]. Nevertheless, children with uCP also display decreased function in their dominant hand compared to TDC, possibly influencing their bimanual function [22]. For this reason, it is critical to have a better understanding of the relation between unimanual performance of both hands during these robotic tasks and bimanual coordination. Lastly, we also expect that bimanual coordination plays a crucial role in the task execution, which reflects the child’s overall performance. However, this relation has not yet been investigated in children with uCP with the Kinarm exoskeleton robot. This information could aid in a more motivated selection of bimanual coordination tasks and limit the assessment time in future studies.

Hence, the aim of this study is threefold. The first aim is to compare bimanual coordination in children with uCP and TDC using three robotic tasks. By including TDC as a control group, we aim to gain insights into the presence of bimanual coordination differences between children with uCP and TDC in order to identify potential bimanual coordination deficits and define sensitive outcome parameters. The second aim was to investigate whether bimanual coordination parameters differ between children with uCP with different manual abilities. Third, we will explore the relationship between unimanual parameters, task execution parameters, and bimanual coordination parameters of different robotic tasks in children with uCP. By combining these insights, we want to gain a better understanding of sensitive tasks and parameters for measuring bimanual coordination in children with uCP using the Kinarm exoskeleton robot. We expect that (1) children with uCP will have bimanual coordination deficits across all three tasks; (2) children with better manual ability will have a better bimanual coordination; (3) that bimanual coordination will be related to task performance and (4) that unimanual parameters of both hands will be linked to both aspects of bimanual coordination, i.e. bimanual coupling and interlimb differences.

## Methods

### Participants

Children with uCP, aged 7–15 years were recruited from the CP care program of the University Hospitals Leuven, Belgium for a larger project involving multiple cross-sectional studies. Children with uCP were included if they could understand the test instructions and if they were able to actively grasp and stabilize an object with the non-dominant hand (House Functional Classification Score ≥ 4) [23]. This last inclusion criteria was related to the overall project, which includes other upper limb assessments that require an active grasp of the non-dominant hand. Children were excluded if they had received botulinum neurotoxin A injections in the upper limb six months before testing or had undergone upper limb surgery two years before assessment. Children with uCP were further categorized according to their manual abilities in levels I to III using the MACS, where a higher level indicates worse manual ability [20]. Individually age- and sex-matched TDC were included when they had no neurological disorder, musculoskeletal problems of the upper limb, or uncorrected visual impairments. Written informed consent was obtained by all parents, and children above the age of 12 also provided their assent to participation. The Ethics Committee Research UZ/KU Leuven authorized the study (S62906), which was carried out in conformity with the Helsinki Declaration of 1964.

### Robotic assessment of bimanual coordination

#### Apparatus and test administration

**Fig. 1:**
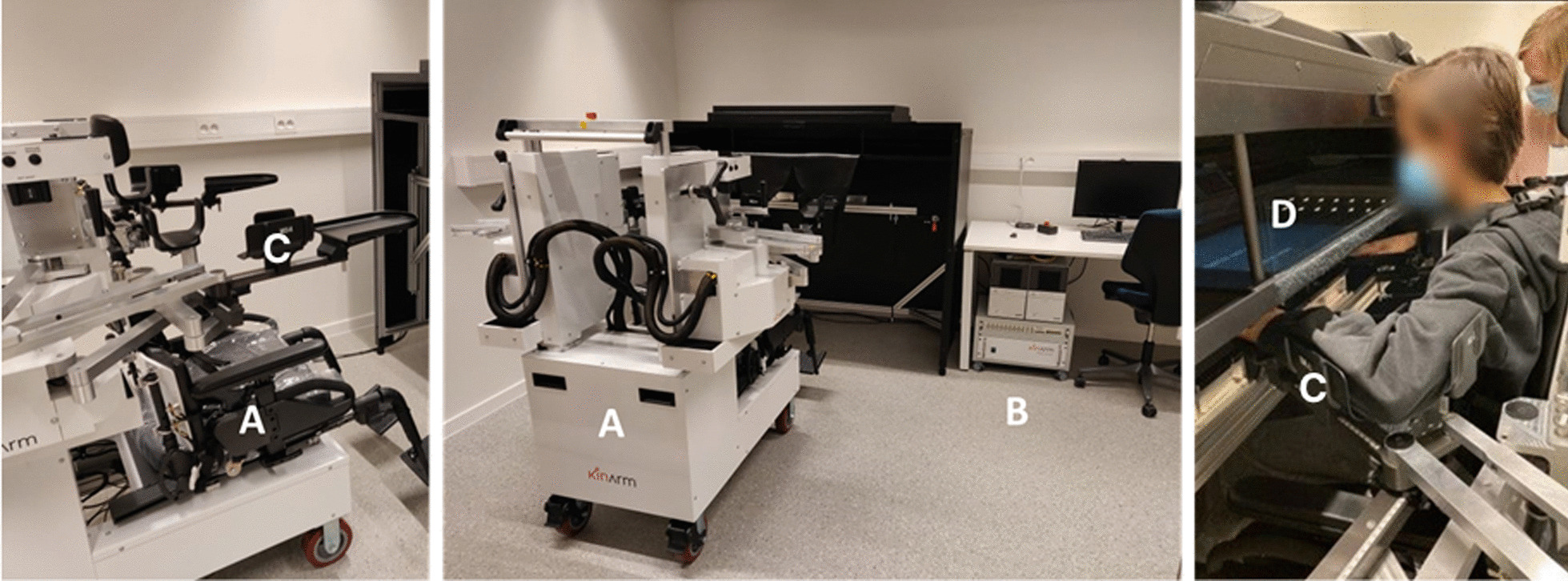
Kinarm exoskeleton robot with the exoskeleton (**A**), computer for data acquisition (**B**), one of the three arm supports (more specifically for the underam) (**C**) and interactive screen (**D**)

The Kinarm robotic exoskeleton (Kinarm, BKIN Technologies Ltd, Kingston, Canada) was used to quantify bimanual coordination (Fig. 1). For the measurements, participants were seated in an adaptable exoskeleton with their arms and legs supported. Younger children who were of insufficient height were positioned on a cushion to lift them to the proper seated position. Each upper limb was placed on three arm supports or troughs, providing support for the entire limb, while the shoulders were placed in 85° shoulder abduction. These supports were individually adjusted to align with the participant’s shoulder and elbow joints, making free movements in the horizontal plane possible. The child was strapped in the chair to limit trunk movements. A mirrored screen displayed the task and cursors that represented the child's hand position in an augmented reality system. During COVID-19, mouth masks were removed to optimize vision once the child was installed in the disinfected area and at a safe distance from the assessors.

#### Bimanual coordination tasks

**Fig. 2:**
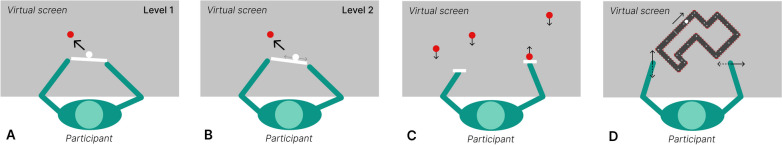
Tasks on the Kinarm exoskeleton: BOB task with level 1 (**A**) and level 2 (**B**), OH task (**C**) and circuit task (**D**)

Three bimanual tasks were assessed: one symmetrical, the ball-on-bar (BOB) task, and two complementary, the object-hit (OH) task and the circuit task (Fig. 2). The BOB and OH tasks are standardized tasks of Kinarm (Collection version 3.9.2, analysis version 3.9.3, BKIN Technologies Ltd., Kingston, ON, Canada) [24], whereas the circuit task was developed by Doost et al. [25]. All tasks were preceded by a practice session to ensure comprehension. Moreover, child-adapted versions of each task were used, which is a scaled-down version of the adult tasks in the horizontal plane, to minimize the extent of the required arm movements.’

The first task was the BOB task [26] (Fig. 2A, B), where participants’ fingertips were connected by a 20 cm virtual white bar on which they had to balance a ball while moving it to a target using both hands. The targets were presented in four positions, one at a time, in a diamond shape with the corners 6 cm from the centre [24]. A spring-like force was applied when either hand moved too far away from their starting position, in order to maintain bar length [26]. The task consists of two difficulty levels, with the ball fixed to the middle of the bar in the first level (Fig. 2A) and the ball moving based on the bar’s tilt in the second level, (Fig. 2B) [13, 15].

In the OH task [27] (Fig. 2C), participants were asked to hit balls falling from the top of the screen using both hands, represented as 3 cm wide lines. Both hands could move freely without restrictions in the horizontal plane. To ascertain bimanual coordination during this task, it was explicitly specified that both hands had to be used. The goal was to hit as many balls as possible in 2 min, while the speed and amount of the balls continuously increased. A total of 300 balls were dropped randomly from 10 different invisible bins within the 50 cm workspace [27].

Lastly, in the circuit task of Doost et al. [25] (Fig. 2D), participants needed to drive a cursor through a circuit as accurately as possible by performing opposite movements with both hands. The left hand moved the cursor only in the vertical direction and the right hand in the horizontal direction. Virtual barriers imposed by the robot ensured that the hands could only move in these directions. The circuit was tilted at a 45° angle relative to the horizontal axis, requesting simultaneous movements of both hands to move the cursor through the oblique paths. The test consisted of two identical circuits that were a mirrored version of each other [25]. In contrast to Doost et al. [25], participants were requested to complete this task at their own pace rather than as quickly as possible, due to the high cognitive demand of the task.

#### Parameters

For each of the standard tasks, outcome parameters were automatically calculated after the assessment and could be divided into task execution, unimanual and bimanual parameters. Thirteen task execution parameters provided information about the overall task performance, 10 unimanual parameters about the performance of each hand separately, and lastly, 19 bimanual parameters about bimanual coordination which could be further divided into bimanual coupling and interlimb differences. The bimanual coupling parameters quantify the extent of how both hands are spatiotemporally coupled, while the interlimb differences are calculated from the differences between the spatiotemporal performance of each hand [12]. The parameters, as well as their classification within performance, unimanual and bimanual (bimanual coupling and interlimb differences) parameters, are described in Table 1. More detailed information can be found in the Kinarm manual (Dexterity-E 3.9) [24]. Since calculations of some bimanual parameters of the OH task (i.e., miss bias, hand transition, hand bias hits, hand speed bias, movement area bias) did not take the hand dominance into account, a multiplication by − 1 was conducted in left-handed children and by 1 in right-handed children to enable comparison between dominant and non-dominant hands [14]. Some interlimb parameters of the BOB task were already calculated using the absolute values of the outcome. Hence, the direction of the asymmetry for these parameters (i.e., reaction time difference, hand speed difference) could not be identified. Furthermore, as the mean bar tilt parameter had a different sign depending on the direction of the angle of the bar, we used absolute values of this parameter for further analysis [26].

**Table 1:** Overview of Kinarm exoskeleton parameters of the three bimanual coordination tasks

Task	Classification	Parameter	Description [24]
Ball-on-bar task (Level 1 and 2)	Task execution	Targets complete	Amount of successfully touched and held targets (without a ball drop in level 2)
		Drops	Number of times the ball fell of the bar in level 2
		Time to target	Mean time to reach a target from the moment it was visible to the moment it was touched (in s)
		Ball speed	Mean ball speed throughout the level (in cm/s)
	Unimanual	DH/NDH speed	Mean hand speed throughout the level (in cm/s)
		DH/NDH speed peaks	Number of the hand's level-specific speed maxima, omitting those at the first and final data points
	Bimanual: coupling	Mean bar tilt	Absolute value of the mean angle of the bar during the level (in radial)
		Bar tilt standard deviation	Standard deviation of the level’s bar angle (in radial)
		Bar length variability	Variation in bar length (in %)
	Bimanual: interlimb	Hand speed difference	Differences between hand speeds divided by the mean of the hand speeds (in %) Formula = $\frac{\Sigma abs({S}_{DH}-{S}_{NDH})}{\mathrm{0,5}*\Sigma abs({S}_{DH}+{S}_{NDH})}$, with S = hand speed
		Hand speed peaks bias	Relative bias in amount of hand speed peaks Formula = $\frac{{HSP}_{DH}-{HSP}_{NDH}}{{HSP}_{DH}+{HSP}_{NDH}}$, with HSP = hand speed peaks
		Hand path length bias	Relative bias in hand path length Formula = $\frac{{HPL}_{DH}-{HPL}_{NDH}}{{HPL}_{DH}+{HPL}_{NDH}}$, with HPL = hand path length
		Reaction time difference	For each successfully completed target in level 1, the mean absolute difference between both hands is calculated (in s). Formula = $abs({RT}_{DH}-{RT}_{NDH})$, with RT = reaction time. This metric is not calculated in level 2 due to the considerable variability in reaction times due to the moving ball
Object-hit Task	Task execution	Target hits	The percentage of balls hit out of the total number of balls (in %)
		Median error	The point during the entire challenge when 50% of all errors were made with regard to the whole task (in %)
		Miss bias	X-value of the position indicating the direction of most errors. This parameter is calculated based on the number of misses in each of the ten bins with their weighted mean (in cm) Formula = $\frac{{\Sigma }_{i=1}^{10}{x}_{i}* {w}_{m,i}}{{\Sigma }_{i=1}^{10} {w}_{m,i}}$, with x_i_ = X-position of the ith bin, w_m,I_ = weight or the number of misses in the ith bin
	Unimanual	Hits DH/NDH	Total number of hits with the dominant / non-dominant hand
		Hand speed DH/NDH	Mean hand speed of each hand during the task (in cm/s)
		Movement area DH/NDH	Movement area of each hand during the task (in cm^2^)
	Bimanual: Coupling	Hand transition	Absolute x-value of the subject’s preference for using one hand over the other in the workspace (in cm). Larger value indicates a more shifted representation of the midline (in cm) Formula = $\left(\frac{{\Sigma }_{i=1}^{10}{x}_{i}* {w}_{h(DH),i}}{{\Sigma }_{i=1}^{10} {w}_{h(DH),i}}+\frac{{\Sigma }_{i=1}^{10}{x}_{i}* {w}_{h(NDH),i}}{{\Sigma }_{i=1}^{10} {w}_{h(NDH),i}} \right)/2$, with x_i_ = x-position of the ith bin, w_h(DH/NDH),i_ = weight or the number of hits of the DH/NDH in the i^th^ bin. [14]
		Hand selection overlap	Percentage that captures effectiveness of using both hands (i.e. hit balls with both the dominant and non-dominant hand) (in %). Calculated by the sum of the hand changes for each bin divided by total number of hits
	Bimanual: Interlimb	Hand bias hits	Bias in hits between hands divided by the total sum of both hands. Formula = $\frac{{H}_{DH}-{H}_{NDH}}{{H}_{DH}+{H}_{NDH}}$, with H = hits
		Movement area bias	Bias in mean movement area between hands divided by the total sum of both hands Formula = $\frac{{A}_{DH}-{A}_{NDH}}{{A}_{DH}+{A}_{NDH}}$, with A = movement area
		Hand speed bias	Bias in mean hand speed between hands divided by the total sum of both hands Formula = $\frac{{S}_{DH}-{S}_{NDH}}{{S}_{DH}+{S}_{NDH}}$, with S = hand speed
Circuit task	Task execution	Error	The average surface between the cursor trajectory and the midline of the track (in cm^2^). [25]
		Velocity	Average vectorial velocity of both hands (in cm/s). [25]
		Skill	Measure of ideal accuracy (lower error) and speed, by dividing the velocity measurement with the error parameter. This value is multiplied by 1 cm/s to eliminate its dimension. Higher skill values are associated with more accurate movements and/or higher movement speed. [25]
	Bimanual	Bimanual coordination factor	Calculated by dividing the minimum value between the absolute values of each hand by the hand’s vectorial velocities. When the child was only using one hand, the score was adjusted by using a penalty. The score ranges from 0 to 0.7, where a lower score indicates a more sequential movement of both hands. [25] Formula = $\frac{\mathrm{min}(\left|{V}_{x}\right|,\left|{V}_{y}\right|)}{\sqrt{{v}_{x }^{2}+{v}_{y }^{2}}}$, with v_x/y_ = absolute value of the horizontal/vertical hand velocity. [25]

*DH*  dominant hand, *NDH*  non-dominant hand, *cm* centimeter, *s*  seconds

### Statistical analysis

Statistical analysis included one-way analysis of covariance (ANCOVA) to compare bimanual parameters between children with uCP and age- and sex-matched TDC with age as covariate. The parameters were transformed if the Shapiro–Wilk test indicated that the residuals from the ANCOVA were not normally distributed. An overview of the transformations can be found in  Additional file 1. In case of transformation, all results were back-transformed and reported in this study for optimal interpretation [28]. Moreover, when unequal variances were present between groups (Levene’s test, p ≤ 0.05), ANCOVA was performed using robust standard errors [29, 30]. If there was no significant interaction between age and group, only the model with the main effects was retained. When an interaction effect was found between group and age, a moderated regression analysis was performed instead of an ANCOVA [31]. Lastly, to correct for multiple testing, a false discovery rate was implemented with an adjusted p-value of 0.05, indicating that only 5% of significant tests will result in false positives [32, 33]. Effect sizes were calculated using partial eta squared (${\eta }_{p}^{2}$) and interpreted as small (0.01–0.059), medium (0.06–0.13), and large (≥ 0.14) [34]. For the second research question, an ANCOVA following the same procedure as described above was used to compare the bimanual parameters between MACS-levels (Levels l–lll) in children with uCP. Finally, the relation between the bimanual, unimanual and task execution parameters was investigated with Pearson (r) and Spearman’s rank correlations (r_s_), depending on the data distribution, and interpreted as no or little (< 0.30), low (0.30–0.49), moderate (0.50–0.69), high (0.70–0.89) and very high (≥ 0.90) [35]. Matlab 2021a (Mathworks, Natick, MA) was used for data analysis of the circuit task and SPSS Statistics version 26.0 (IBM, Armonk, New York, USA) was used for all statistical analyses. We further used the PlotsOfData application to visualize the difference in bimanual coordination between children with different MACS-levels. [36]

## Results

### Participants

In this study, 50 children with uCP (11y11m ± 2y10m, 27 males, 27 right-sided uCP) and 50 individually age- and sex-matched TDC (11y11m ± 2y10m, 44 right-handed) were included. Children with uCP were further classified according to their MACS-level (MACS-levels: l: N = 27, ll: N = 16, lll: N = 7). Technical problems caused missing data in some parameters. These children and their match were omitted from the analysis of the specific parameter in the comparison between children with uCP and TDC. Data analysis was conducted on the first level of the BOB and OH tasks using 50 matches, except for the BOB task's reaction time difference parameter (47 matches; MACS-levels: l: N = 27, ll: N = 16, lll: N = 6) and the OH task's hand speed bias (49 matches; MACS-levels: l: N = 27, ll: N = 16, lll: N = 7). The second level of the BOB task was analysed with 48 matches (MACS-levels: l: N = 26, ll: N = 16, lll: N = 7) and the circuit task with 46 matches (MACS-levels: l: N = 26, ll: N = 16, lll: N = 5). A full overview of the total number of included participants for each parameter can be found in  Additional file 2.

### Comparison of bimanual parameters between children with uCP and TDC

**Fig. 3:**
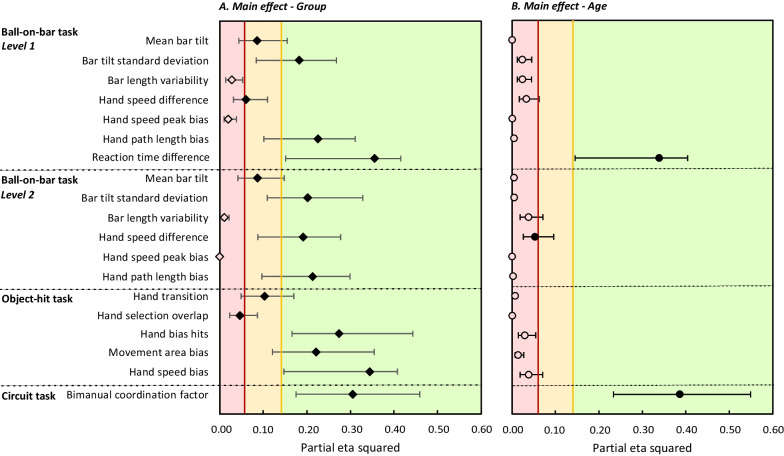
Effect sizes (partial eta squared) with their 90% confidence interval from the ANCOVA investigating the difference in bimanual coordination between uCP and TDC for the main effect of group (**A**) and age (**B**). Partial eta squared is classified and presented in red (low effect size), yellow (medium effect size) and green (large effect size). A filled symbol (diamond or circle) represents a significant difference with p ≤ 0.05

First, we investigated the difference in bimanual coordination parameters of the three tasks between children with uCP and TDC. A visual representation of the effect sizes from the ANCOVA can be found in Fig. 3 and an overview of the descriptives of the ANCOVA with mean and 95% confidence interval shown in Table 2.

**Table 2:** Overview of ANCOVA of bimanual parameters between children with uCP and TDC

Parameters	Mean (95% CI)		Grouping	Age
	TDC	uCP	P* (η^2^_p_)	P* (η^2^_p_)
*Ball-on-bar task: Level 1*				
Mean bar tilt (Rad,↓)	0.02 (0.02–0.04)	0.06 (0.04–0.08)	**0.01 (0.09)**	0.95 (0.00)
Bar tilt standard deviation (Rad,↓)	0.07 (0.07–0.09)	0.12 (0.10–0.15)	** < 0.001 (0.18)**	0.17 (0.02)
Bar length variability (%,↓)	3.40 (3.04–3.77)	3.84 (3.47–4.24)	0.14 (0.03)	0.17 (0.02)
Hand speed difference (%,↓)	43.32 (40.93–45.86)	47.82 (45.19–50.61)	**0.03 (0.06)**	0.11 (0.03)
Hand speed peak bias (#/#,↓)	0.03 (0.02–0.04)	0.04 (0.03–0.06)	0.20 (0.02)	0.86 (0.00)
Hand path length bias ($\frac{\mathrm{cm}}{\mathrm{cm}}$,↓)	− 0.02 (− 0.03–(− 0.01))	0.03 (0.01–0.05)	** < 0.001 (0.23)**	0.56 (0.00)
Reaction time difference (s, ↓)	0.04 (0.04–0.05)	0.07 (0.06–0.08)	** < 0.001 (0.36)**	** < 0.001 (0.34)**
*Ball-on-bar task: Level 2*				
Mean bar tilt (Rad,↓)	0.02 (0.01–0.02)	0.03 (0.03–0.04)	**0.01 (0.09)**	0.58 (0.00)
Bar tilt standard deviation (Rad,↓)	0.05 (0.05–0.06)	0.07 (0.07–0.08)	** < 0.001 (0.20)**	0.57 (0.01)
Bar length variability (%,↓)	3.09 (2.73–3.51)	3.39 (2.99–3.83)	0.38 (0.01)	0.09 (0.04)
Hand speed difference (%,↓)	40.37 (38.63–42.20)	48.03 (44.98–51.29)	** < 0.001 (0.19)**	**0.04 (0.05)**
Hand speed peak bias (#/#,↓)	0.03 (0.01–0.04)	0.03 (0.01–0.04)	1.00 (0.00)	1.00 (0.00)
Hand path length bias ($\frac{\mathrm{cm}}{\mathrm{cm}}$, ↓)	− 0.03 (− 0.04–(− 0.02))	0.02 (0.00–0.04)	** < 0.001 (0.21)**	0.73 (0.00)
*Object-hit task*				
Hand transition (m, ↓)	− 0.01 (− 0.02–0.00)	− 0.03 (− 0.04–(− 0.02))	** < 0.001 (0.10)**	0.90 (0.00)
Hand selection overlap (-, ↑)	0.11 (0.09–0.12)	0.13 (0.11–0.14)	**0.05 (0.05)**	0.09 (0.04)
Hand bias hits (#/#, ↓)	0.06 (0.04–0.08)	0.19 (0.15–0.23)	** < 0.001 (0.27)**	0.49 (0.01)
Movement area bias ($\frac{{{\text{cm}}^{2} }}{{{\text{cm}}^{2} }}$,↓)	0.00 (-0.05–0.05)	0.19 (0.14–0.23)	** < 0.001 (0.22)**	0.14 (0.03)
Hand speed bias ($\frac{\mathrm{cm}/\mathrm{s}}{\mathrm{cm}/\mathrm{s}}$, ↓)	0.02 (0.00–0.05)	0.19 (0.13–0.24)	** < 0.001 (0.34)**	0.31 (0.01)
*Circuit task*				
Bimanual coordination factor (arb. unit,↑)	0.31 (0.30–0.32)	0.26 (0.25–0.27)	** < 0.001 (0.31)**	** < 0.001 (0.39)**

*ANCOVA*  analysis of covariance, *TDC*  typically developing children, *uCP*  unilateral cerebral palsy, *CI*  confidence interval, P-value* = adjusted P-value with false discovery rate of 0.05 for multiple comparison, η^2^_p_ = partial eta square, ↑ = higher value is better bimanual coordination, ↓ = lower value is better bimanual coordination, bold = significant p-value ≤ 0.05

#### Ball-on-bar task (BOB)

The results of the BOB task showed that children with uCP had worse bimanual coupling compared to TDC. Significant between group differences were found in two bimanual coupling parameters, with a moderate (mean bar tilt; level 1 and 2: p = 0.01, $\eta_{{\text{p}}}^{2}$ =0.09) to high effect size (bar tilt standard deviation; level 1: p < 0.001, $\eta_{{\text{p}}}^{2}$ =0.18; level 2: p < 0.001, $\eta_{{\text{p}}}^{2}$ =0.20). No significant difference with only a small effect size was found for bar length variability (level 1: p = 0.14, $\eta_{{\text{p}}}^{2}$ =0.03; level 2: p = 0.38, $\eta_{{\text{p}}}^{2}$ =0.01). For interlimb differences, reaction time difference from the first level, hand speed difference from the second level and hand path length bias from both levels showed significant differences between groups with a large effect size, indicating greater differences between hands in children with uCP compared to TDC (level 1—reaction time difference: p < 0.001, $\eta_{{\text{p}}}^{2}$ =0.36; level 2—hand speed difference: p < 0.001, $\eta_{{\text{p}}}^{2}$ =0.19; both levels—hand path length bias: p < 0.001, $\eta_{{\text{p}}}^{2}$ =0.21–0.23). Interlimb differences in hand path length are presented by higher path length of the dominant hand in children with uCP, whereas TDC exhibited longer hand path for their non-dominant hand (Fig. 5). Hand speed peak bias did not show a significant difference between groups with only a small effect size (p = 0.20–1.00, $\eta_{{\text{p}}}^{2}$ =0.00–0.02). Regarding the effect of age, a main effect was found for reaction time difference, with a large effect size (p < 0.001, ${\eta }_{p}^{2}$=0.34, Fig. 3B) and hand speed difference with a small effect size (p = 0.04, ${\eta }_{p}^{2}$=0.05, Fig. 3B), indicating that older children exhibited less differences between hands for both groups.

#### Object-hit task (OH)

Bimanual coupling parameters of the OH task showed a significant difference between groups, but only with a low to medium effect size (hand transition: p < 0.001, ${\eta }_{p}^{2}$=0.10; hand selection overlap: p = 0.05, ${\eta }_{p}^{2}$=0.05), indicating that children with uCP crossed the workspace further with their dominant hand and required more space to use both hands effectively compared to TDC. Interlimb differences in the OH task showed that children with uCP hit more balls, moved more and faster with their dominant hand compared to their non-dominant hand, illustrated by large effect sizes (hand bias hits: p < 0.001, ${\eta }_{p}^{2}$=0.27; movement area bias: p < 0.001, ${\eta }_{p}^{2}$=0.22; hand speed bias: p < 0.001, ${\eta }_{p}^{2}$=0.34), resulting in more differences between hands in comparison to TDC. No significant age effects were found with only low effect sizes for the bimanual parameters of the OH task (p = 0.14–0.90; ${\eta }_{p}^{2}$=0.00–0.04).

#### Circuit task

The circuit task showed a significant difference in bimanual coupling between groups with a large effect size (bimanual coordination factor: p < 0.001, ${\eta }_{p}^{2}$=0.31), showing that children with uCP performed the circuit task more sequentially compared to TDC. A significant influence of age was found for this parameter with a large effect size, showing that better coordination was present in older children in both groups (p < 0.001, ${\eta }_{p}^{2}$=0.39).

### Comparison of bimanual parameters between MACS-levels

**Fig. 4:**
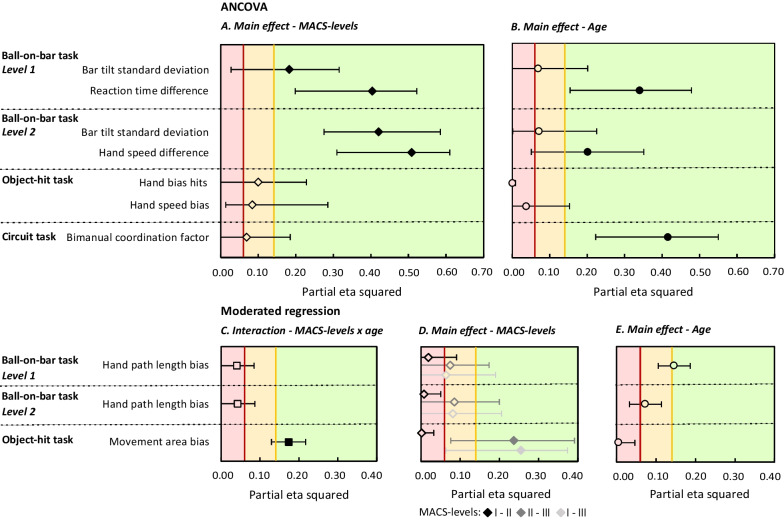
Effect sizes (partial eta squared) with their 90% confidence interval from the ANCOVA (**A**, **B**) and moderated regression (**C**–**E**) investigating the difference in bimanual coordination in children with uCP across different MACS-levels for the selection. For the ANCOVA, the effect sizes for the main effect of MACS-levels (**A**) and age (**B**) is presented. Effect sizes are shown for the moderated regression for the interaction between MACS-levels and age (**C**) and main effect of MACS-levels (**D**) and age (**E**). Partial eta squared is classified and presented in red (low), yellow (medium) and green (large). A filled symbol (diamond, circle or square) represents a significant difference with a p ≤ 0.05

**Fig. 5:**
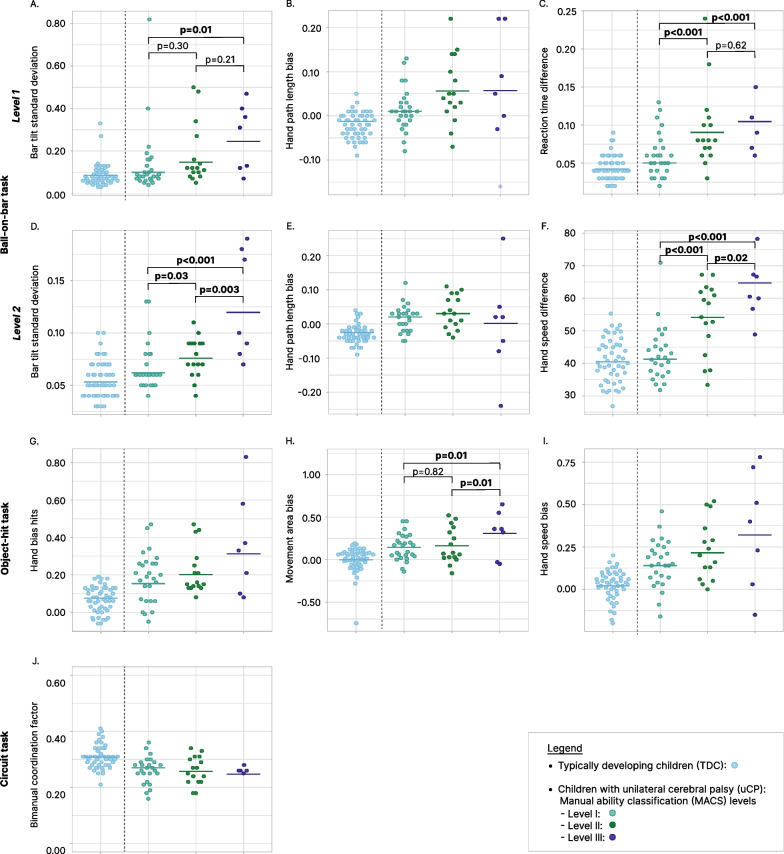
Individual data points with back-transformed mean for the parameters of the ball-on-bar (**A**–**F**), object-hit (**G**–**I**) and the circuit task (**J**). Results of the differences between MACS-levels in children with uCP is presented in solid lines if a significant main effect of MACS-levels was present. *TDC*  typically developing children (blue), *uCP*  unilateral cerebral palsy, *MACS*  manual classification system (level l = turquoise, level ll = green, level lll = purple)

We selected the parameters that exhibited large differences between children with uCP and TDC, with the goal of prioritizing parameters that reflect reduced bimanual coordination in children with uCP. This selection resulted in three bimanual coupling parameters (BOB and circuit task) and seven interlimb difference parameters (BOB and OH task). Figure 4 provides a summary of the effect sizes for this selection, while Fig. 5 presents the individual data points with post-hoc comparison. Due to three parameters (BOB task level 1 and 2: hand path length bias, OH task: movement area bias) exhibiting an interaction effect with age, a moderate regression analysis was conducted, leading to two separate analyses (ANCOVA and moderated regression) in this objective. Tables with the descriptive statistics of all the parameters are provided in the Additional files 3, 4, 5.

#### Ball-on-bar task (BOB)

The BOB task showed that for the bimanual coupling parameters, the bar tilt standard deviation of both levels, were influenced by the MACS-levels with high effect sizes. This indicates that children with lower manual ability or MACS level lll presented greater variability in bar tilt or reduced bimanual coupling (bar tilt standard deviation; level 1: p = 0.02,${ \eta }_{p}^{2}$=0.18; level 2: p < 0.001, ${\eta }_{p}^{2}$=0.36), compared to children with MACS l (p = 0.01) in level 1 and to MACS l (p < 0.001) and ll (p < 0.02) in level 2. For interlimb differences, level 1 of the BOB task demonstrated less difference between hands for reaction time with a high effect size (p < 0.001, ${\eta }_{p}^{2}$=0.40) in MACS I compared to MACS II (p < 0.001) and MACS III (p < 0.001). No difference was found between children with a MACS level ll and lll (p = 0.62). Moreover, moderated regression indicated no moderation of age and no significant differences in hand path length bias between MACS-levels for both levels of the BOB task with only low effect sizes (interaction: p = 0.37, ${\eta }_{p}^{2}$=0.04; main effect MACS-levels: p = 0.15–0.67, ${\eta }_{p}^{2}$=0.01–0.08; main effect age: p = 0.05–0.19, ${\eta }_{p}^{2}$=0.07–0.15). For the second level of the BOB task, hand speed difference was significantly different between groups with a high effect size (p < 0.001,${ \eta }_{p}^{2}$=0.51), indicating that children with a MACS-level lll showed more difference in speed between hands compared to MACS l and ll (MACS I-II-III: p ≤ 0.02).

#### Object-hit task (OH)

For the OH task, the hand bias hits and hand speed bias did not show a significant difference between MACS-levels, despite moderate effect sizes (hand bias hits: p = 0.15, ${\eta }_{p}^{2}$=0.10; hand speed bias: p = 0.20, ${\eta }_{p}^{2}$=0.12). Figure 5G and I show a trend indicating that children with lower manual ability displayed greater interlimb differences. Lastly, moderated regression indicated that age significantly moderated the difference in OH task movement area bias across MACS-levels with a high effect size (interaction: p = 0.01, ${\eta }_{p}^{2}$=0.16). This means that interlimb differences in movement area increased with age in children with MACS-level lll, while they decreased with age in children with MACS level I (b = − 0.04, t = − 3.13, p = 0.003). No relation with age was found in children with MACS level II (b = − 0.04, t = − 2.94, p = 0.01) compared to children with MACS lll. Furthermore, no moderating effect of age (b = 0.00, t = − 0.35, p = 0.71, ${\eta }_{p}^{2}$=0.006) and no significant difference was found between MACS-levels l and ll (b = 0.02, t = 0.22, p = 0.82, ${\eta }_{p}^{2}$=0.002), with only small effect sizes.

#### Circuit task

The bimanual coordination factor of the circuit task showed no significant difference between MACS-levels with a moderate effect size (p = 0.32,${ \eta }_{p}^{2}$=0.07).

### Relation between bimanual and unimanual or task execution parameters

**Fig. 6:**
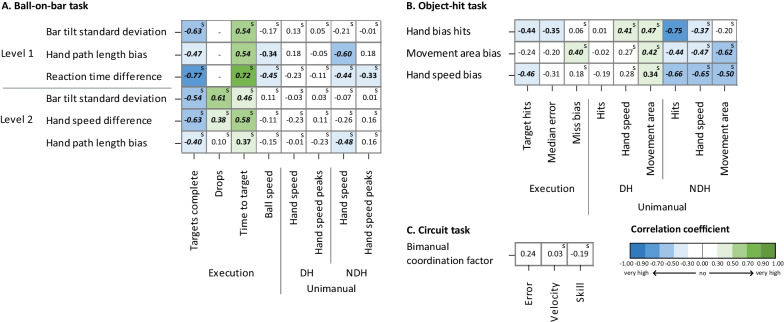
Pearson and spearman's rank correlation coefficients of the unimanual and task execution parameters with the bimanual parameters of the ball-on-bar task (**A**), object-hit task (**B**) and circuit task (**C**) for the selected parameters in children with uCP. *DH*  dominant hand, *NDH*  non-dominant hand, *s*  spearman’s rank correlation

Similar to previous objective, we utilized the same selection of parameters to emphasize those that exhibit a large difference between uCP and TDC. An overview of the correlation coefficients between the selected bimanual and unimanual and task execution parameters in children with uCP can be found in Fig. 6. Correlation matrix including all parameters can be found in additional file Additional file 6.

#### Relation between bimanual and task execution parameters

*For the BOB task*, bimanual coupling was moderately related with the task execution parameters, showing that higher bar tilt standard deviation was associated with a lower number of completed targets in both levels (r_s_ = − 0.54–(− 0.63), p < 0.001), slower time to target (r_s_ = 0.54, p < 0.001) in level 1 and more drops in level 2 (r_s_ = 0.61, p < 0.001). More interlimb differences in path length between hands of children with uCP was moderately associated with a higher time to target in level 1 (r = 0.54, p < 0.001) and more difference in speed between both hands was moderately related to a lower number of completed targets (r_s_ = − 0.63, p < 0.001) and a higher time to target (r_s_ = 0.58, p < 0.001) in level 2. Lastly, more difference in reaction time between hands was highly related to reaching less targets (targets complete, r_s_ = − 0.77, p < 0.001) and needing more time to reach the target (time to target, r_s_ = 0.72, p < 0.001) in level 1.

For the interlimb differences of the *OH task* and bimanual coupling parameter of the *circuit task*, only no to low correlations were found (r and r_s_ = − 0.46–0.44, p = 0.001–0.96).

#### Relation between bimanual and unimanual parameters

For bimanual coupling of the *BOB task*, no correlations were found between the bar tilt standard deviation and the unimanual parameters of both hands in both levels (r_s_ = − 0.21–0.13, p = 0.15–0.95). Hand speed of the non-dominant hand was moderately associated to hand path length bias in the first level of the BOB task (r = − 0.60, p < 0.001) and lowly related in the second level (r_s_ = − 0.48, p < 0.001), showing that a higher speed of the non-dominant hand was related to less interlimb differences for hand path length. Also, higher speed and more speed peaks of the non-dominant hand were only lowly related to less reaction time difference in the first level of the BOB task (r_s_ = − 0.44–(− 0.33), p = 0.004–0.03).

*For the OH task*, the parameter hand bias hits was highly related to the hits of the non-dominant hand (r = − 0.75, p < 0.001) and movement area bias to movement area of the non-dominant hand (r_s_ = − 0.62, p < 0.001), respectively. Hand speed bias was moderately correlated with all unimanual parameters of the non-dominant hand, where more interlimb asymmetry in hand speed was related to fewer hits (r = − 0.66, p < 0.001), slower hand speed (r_s_ = − 0.65, p < 0.001) and less movement area (r_s_ = − 0.50, p < 0.001) of the non-dominant hand.

## Discussion

Our findings indicate that children with uCP demonstrate impaired bimanual coordination, which can be established using the Kinarm exoskeleton robot, as shown by increased bimanual coupling deficits (BOB and circuit task) and interlimb differences (BOB and OH task) compared to TDC. Interestingly, some of these impairments showed improvement as children get older, but the difference between groups remained the same. Our results also demonstrated that bimanual coordination was associated with manual ability, particularly for the BOB and OH tasks, showing that a worse manual ability was related to worse bimanual coordination. Furthermore, moderate to high correlations were found in children with uCP between bimanual coordination and the BOB task execution parameters. In the OH task, greater interlimb differences were mainly linked to a worse non-dominant hand performance.

Our first aim was to investigate differences in bimanual coordination between children with uCP and TDC. For the BOB task, children with uCP showed both bimanual coupling deficits and more interlimb differences in both levels compared to TDC. These results are similar to the results found in the adult stroke population, except that adult stroke patients showed a greater number of impaired parameters of bimanual coordination in the first level of the BOB compared to the second level [26]. Although research investigating bimanual coordination using a symmetrical tray lifting showed that more difficult task demands, which is represented in the second level of the BOB task by the ball's motion, could lead to better bimanual coordination in children with uCP, it also showed that it results in more compensational strategies [37]. This reasoning, together with the fact that children have a shorter attention span compared to adults [38], could explain why children with uCP have similar bimanual coordination deficits in the second level of the BOB task compared to the first level and compared to adult stroke patients. Only hand speed difference showed a larger difference between groups in the second level of the BOB task compared to the first level, as TDC showed less interlimb difference whereas children with uCP more. This is an example that higher task demands lead to improved bimanual coordination in TDC, whereas it decreases in children with uCP possibly due to compensatory movement required to prevent the ball from falling off the bar when the bar is tilted. Second, for the OH task, Hawe et al. found significant interlimb differences in children with uCP compared to TDC [14], which is consistent with our results. Hence, we may conclude that the OH task primarily measures interlimb differences. This is not unexpected, since both hands can independently execute the task, limiting the amount of bimanual coupling. Lastly, the results of the circuit task revealed that children with uCP move both hands more sequentially in bimanual tasks than TDC. This was evidenced by a notably high effect size, indicating worse bimanual coupling in children with uCP. This is consistent with other studies, investigating bimanual coordination with an asymmetric drawer opening task, showing that children with uCP complete bimanual tasks more consecutively [11]. A possible explanation could be the necessity of two contrasting components when performing a bilateral complementary task: the spatiotemporal coupling of both hands to function as a single unit and the neural inhibition of the other hand [39]. In children with uCP, the latter may be disrupted due to the presence of mirror movements, which are unintentional movements in one hand that mirror the voluntary movements of the other hand [40]. Lastly, both the BOB and circuit task presented a main effect of age with a large effect size, showing that older children with uCP and TDC presented better bimanual coordination. This is in line with the study of Hawe et al. who identified an age effect for interlimb differences and bimanual coupling, but only for TDC [14]. Our results are interesting as they demonstrate an improvement in bimanual coordination in older children with uCP, yet the differences between them and TDC persist. Nevertheless, longitudinal studies are needed to confirm these findings.

The second aim examined bimanual coordination differences across children with uCP with different levels of manual ability. In literature, this has only been investigated using a 3D motion analysis of a box opening task, where children with MACS-level lll performed slower, while no variations in bimanual coupling between children with different MACS-levels were found [13]. However, our results showed that children with uCP with worse manual abilities (MACS-level lll) also have poorer bimanual coordination, in particular prominent in the BOB task. Both bimanual coupling and interlimb differences were observed, with the second level of the BOB task being more effective in distinguishing between all three groups, while the first level only had the ability to differentiate MACS l from MACS ll and MACS lll. This is possibly explained by the fact that the second level of the BOB task is more challenging and thus creates more differences between MACS-levels. The OH task also identified interlimb differences in movement area between MACS-levels with a moderated influence of age. Surprisingly, the circuit task showed a moderate effect size between MACS-levels, yet the result was not significant. The small number of participants in the MACS-level lll group, resulting in less power, and possibly impaired executive function in each MACS-level [41], could pose a challenge in finding a significant effect. However, despite these interesting results, we expected more differences between the MACS-levels to emerge. Performance of all tasks could also be influenced by other factors such as impairments in rapid motor planning [41], somatosensory or visual deficits [42] and impaired cognition [43], which could create more variance between participants (Fig. 5) and result in less differences between subgroups of children with uCP. Further research is needed to investigate these differences with a larger sample of children with a MACS-level lll and including the influence of co-morbidities on bimanual coordination. Lastly, to the best of our knowledge, our study is the first to indicate a main effect of age for bimanual coordination in children with uCP. We observed a trend  that children with a MACS-level l or ll present less interlimb difference (BOB task) and better bimanual coupling (circuit task) with increasing age, while children with a MACS level lll were not or less influenced by age. The outcome is not unexpected, given that the deficits observed in children with a MACS level lll may be too severe to exhibit improvement with increasing age.

For the third aim, we investigated the relationship between bimanual parameters and parameters of task execution on the robotic tasks in children with uCP. The BOB showed that worse bimanual coupling and more interlimb differences are related to a worse task execution in both levels. On the other hand, both the bimanual coordination parameters of the OH and circuit task showed no to low correlations with the task execution parameters. These findings indicate that the BOB task most effectively assesses bimanual coordination skills, which is also related to task performance. In contrast, the OH task is less impacted by bimanual coordination difficulties as it can be done with one hand. For the circuit task, a possible explanation could be that the cognitive demands may have a greater impact on task execution than bimanual coordination as explained earlier. Moreover, the present study investigated the relation between unimanual parameters of each hand and bimanual coordination. Our initial hypothesis was that bimanual coordination would be related to unimanual performance of both hands. However, our findings indicate that in children with uCP, interlimb differences were mainly related to the function of the non-dominant hand, specifically in the OH task. This supports the prior belief that the OH task is less connected to using both hands together and is instead more related to the use of the non-dominant hand. For the BOB task, most bimanual coordination parameters showed no to weak correlations with unimanual function of both hands, indicating that there is only minimal connection between the unimanual and bimanual parameters. As the BOB task is a clear measure of bimanual coordination, the absent correlation between unimanual and bimanual parameters supports the theory that bimanual coordination is more complicated than the simple addition of the two hands' capabilities [2]. Other factors like the impairment of neural communication between the two sides of the brain, or structural damage to the corpus callosum, may be more significant factors in explaining limitations in bimanual coordination [39, 44]. One study has found that a better corpus callosum integrity, in particular the splenium region, was associated with an improved bimanual coupling in children with uCP [20]. Nevertheless, more research is needed to fully understand the neuropathophysiology of bimanual coordination deficits in children with uCP. Lastly, it is important to indicate that the unimanual parameters of the Kinarm exoskeleton robot only capture task-specific aspects of their unimanual performance during the execution of bimanual tasks and thus not measure their full unimanual capacity. Nevertheless, it allowed for a direct comparison between bimanual and unimanual performance within the same task context, providing valuable insights into the relative contributions of each hand to bimanual coordination. Although our study provided a first insight into the relation between both hands and bimanual coordination, more research including different aspects of their unimanual capacity is needed.

This study also has some limitations. One of the limitations is the fact that in total, a large number of bimanual parameters were included in the statistical analysis. Nevertheless, to account for multiple testing, a false discovery rate was implemented. A second limitation is the different number of children in the MACS-levels, where only seven children with MACS-level lll were included. However, based on population-based research, it has been shown that only 8% of children with uCP are categorized in MACS-level lll compared to 55% in MACS l and 32% in MACS ll [45]. Thus, our sample represents the actual proportion of MACS levels in the population of children with uCP (54% MACS I, 32% MACS II, 14% MACS III). However, despite the accurate representation of MACS levels within our population, we only included children with uCP who had the ability to actively grasp and stabilize an object with their non-dominant hand. Therefore, our results should not be generalized to the entire population and should be interpreted with caution. Lastly, the Kinarm robot does not provide clear criteria for the inclusion of children, except that a booster seat should be used for younger children. Nonetheless, based on a small pilot study, we observed that only children from the age of seven years onwards appeared to have sufficient height to perform the tasks on the Kinarm. Furthermore, there are no specific inclusion criteria for children with uCP, but we did not encounter any additional difficulties in this study sample.

Notwithstanding its limitations, this study is the first assessing bimanual coordination in a large sample of children with uCP using multiple bimanual tasks with different requirements on a robotic device. In addition, this study offers an initial perspective on the selection of important tasks and parameters when using the Kinarm exoskeleton robot for the assessment of bimanual coordination in children with uCP. Overall, the symmetrical BOB task seems to be the most effective and discriminative task for assessing both interlimb differences and bimanual coupling in children with uCP. On the other hand, the OH task primarily reflects the interlimb differences and the circuit task the bimanual coupling deficits in children with uCP. However, in order to draw final conclusions about the crucial parameters, it is necessary to investigate the impact of bimanual motor control deficits on the functional performance of children with uCP. Although the Kinarm exoskeleton provides quantitative information about bimanual coordination, the movements are still limited to the horizontal plane with possibly low ecological validity. Despite the fact that the results of the BOB were complementary to the more functional 3D motion analysis of a tray lifting task, indicating bimanual coupling deficits in children with uCP [37], more research is needed investigating the relation between robotics and functional measures of bimanual function. This further investigation can validate the current hypothesis in literature regarding the inability of clinical tools, like the AHA, in assessing bimanual coordination [8, 9]. Additionally, it can provide insights into the degree to which the Kinarm exoskeleton complements other assessments of bimanual function. Therefore, although our research is a promising initial starting point, additional investigation is warranted to explore the relationship between robotics, clinical tests, and daily life performance to better understand the added value of robotics and the impact of bimanual coordination deficits in this population.

## Conclusion

To the best of our knowledge, this is the first study that extensively mapped bimanual coordination using different nonpreferred coordination tasks with a robotic device investigating bimanual coordination in children with uCP and TDC. It showed that the Kinarm exoskeleton robot can be used to identify bimanual coupling deficits and interlimb differences in children with uCP, both in bilateral symmetrical and complementary tasks. Moreover, mainly the symmetrical BOB task was able to differentiate between children with uCP and varying levels of manual ability, showing children with lower manual abilities have more bimanual coordination deficits. Moreover, the coordination deficits measured with the BOB task also showed a relation with the task execution. Additionally, it appears that the bilateral complementary OH and circuit tasks are able to assessing distinct aspects of bimanual coordination. The OH task mainly provides more information about the larger interlimb differences in children with uCP, which are mainly associated with diminished functionality of the non-dominant hand. Furthermore, the circuit task provided information about the bimanual coupling deficits in children with uCP showing equal limitation in children with uCP with different manual abilities. Future research should focus on the impact of coordination deficits on the performance of daily life activities in children with uCP to further define the merit of robotics in assessing bimanual coordination and investigate possible neurological correlates to understand the neuropathophysiology of the bimanual coordination deficits.

### Supplementary Information

**Additional file 1.** Overview of transformation of the bimanual parameters of the Kinarm exoskeleton. Used transformations of bimanual parameters for the analysis of covariance between children with uCP and TDC and between children with uCP with different MACS levels.

**Additional file 2.** Overview of the number of children in each ANCOVA analysis. A full overview of the total number of included participants for the analysis of covariance between children with uCP and TDC and between children with uCP with different MACS levels.

**Additional file 3.** Effect sizes of the differences in bimanual coordination between different MACS-levels for all bimanual parameters. Effect sizes (partial eta squared) with their 90% confidence interval from the difference in bimanual coordination in children with uCP with different MACS-levels. For the ANCOVA (A,B): the effect sizes for the main effect of MACS-levels (A) and age (B) is presented. Effect sizes are shown for the moderated regression (C-E) for the interaction between MACS level and age (C), main effect of MACS-level (D) and age (E). Partial eta squared is classified and presented in red (low). yellow (medium) and green (large). A filled in symbol (diamond, circle or square) represent a significant difference with a p ≤ 0.05. MACS = manual ability classification system.

**Additional file 4.** Overview of ANCOVA of bimanual parameters between MACS-levels in children with uCP. A full overview of the results of the analysis of covariance of the bimanual parameters in children with uCP with different manual ability classification levels (MACS-levels). uCP = unilateral cerebral palsy, SD = standard deviation, MACS = manual ability level, P-value* = adjusted P-value with false discovery rate of 0.05 for multiple comparison, η^2^p = partial eta square, bold = significance p ≤ 0.05*.*

**Additional file 5.** Overview of moderated regression of bimanual parameters between MACS_levels in children with uCP. A full overview of the results of the moderated regression of the bimanual parameters in children with uCP with different manual ability classification levels (MACS-levels). MACS = manual ability level, uCP = unilateral cerebral palsy, BOB = Ball-on-bar task, OH = object-hit task, p = p-value with significance ≤ 0.05, R^2^ = effect size, B = coefficient, t = t-value, bold = significance p ≤ 0.05.

**Additional file 6.** Correlation coefficients of all unimanual and task execution parameters with the bimanual parameters. Pearson and spearman's rank correlation coefficients of the unimanual and task execution parameters with the bimanual parameters of the ball-on-bar task (A), object-hit task (B) and Circuit task (C) for the selected parameters in children with uCP. DH = dominant hand, NDH = non-dominant hand, s = spearman’s rank correlation.

## Data Availability

All data concerning this study is available within the manuscript. Detailed data is available upon reasonable request to the first author.

## Abbreviations

uCP: Unilateral cerebral palsy
TDC: Typically developing children
MACS: Manual ability classification system
BOB task: Ball-on-bar task
OH task: Object-hit task
