# Supplementary material for: In-depth quantification of bimanual coordination using the Kinarm exoskeleton robot in children with unilateral cerebral palsy
Source: J Neuroeng Rehabil. 2023 Nov 11;20:154. doi: 10.1186/s12984-023-01278-6 (PMC10640737; doi:10.1186/s12984-023-01278-6)
Supplement: Supplementary file 1 — Additional file 1. Overview of transformation of the bimanual parameters of the Kinarm exoskeleton. Used transformations of bimanual parameters for the analysis of covariance between children with uCP and TDC and between children with uCP with different MACS levels. [file 12984_2023_1278_MOESM1_ESM.pdf]

Additional file 1: Overview of transformation of the bimanual parameters of the Kinarm exoskeleton

Task	Classification	Parameter	Transformation: Comparison uCP - TDC	Transformation: Comparison MACS levels
BOB task	Bimanual: coupling	***Mean bar tilt***	Level 1: logarithm,<br>Level 2: square root	Level 1: logarithm,<br>Level 2: square root
		Bar tilt standard deviation	Level 1: logarithm,<br>Level 2: logarithm	Level 1: logarithm,<br>Level 2: logarithm
		Bar length variability	Level 1: square root,<br>Level 2: logarithm	Level 1: square root,<br>Level 2: logarithm
	Bimanual: interlimb	***Reaction time difference***	Level 1: logarithm	Level 1: logarithm
		Hand speed difference	Level 1: logarithm,<br>Level 2: logarithm	Level 1: logarithm,<br>Level 2: none
		Hand speed peaks bias	Level 1: none,<br>Level 2: none	Level 1: none,<br>Level 2: none
		Hand path length bias	Level 1: none,<br>Level 2: reflect logarithm	Level 1: none,<br>Level 2: none
OH task	Bimanual: Coupling	***Hand transition***	none	none
		Hand selection overlap	square root	square root
	Bimanual: Interlimb	***Hand bias hits***	logarithm	logarithm
		Hand speed bias	none	square root
		Movement area bias	reflect logarithm	reflect logarithm
Circuit task	Bimanual	***Bimanual coordination factor***	none	None

BOB task = Ball-on-bar task, OH task = Object hit task, uCP = unilateral cerebral palsy, TDC = typically developing children, MACS = manual Ability Classification System
