# Supplementary material for: In-depth quantification of bimanual coordination using the Kinarm exoskeleton robot in children with unilateral cerebral palsy
Source: J Neuroeng Rehabil. 2023 Nov 11;20:154. doi: 10.1186/s12984-023-01278-6 (PMC10640737; doi:10.1186/s12984-023-01278-6)
Supplement: Supplementary file 2 — Additional file 2. Overview of the number of children in each ANCOVA analysis. A full overview of the total number of included participants for the analysis of covariance between children with uCP and TDC and between children with uCP with different MACS levels. [file 12984_2023_1278_MOESM2_ESM.pdf]

Additional file 2: Overview of the number of children in each ANCOVA analysis

Task	Parameter	Comparison uCP - TDC		Comparison MACS-levels		
		**uCP (n)**	**TDC (n)**	**Level I (n)**	**Level II (n)**	**Level III (n)**
**BOB task – Level 1**	**Mean bar tilt**	50	50	27	16	7
	**Bar tilt standard deviation**	50	50	27	16	7
	**Bar length variability**	50	50	27	16	7
	**Reaction time difference**	***47***	***47***	27	16	***6***
	**Hand speed difference**	50	50	27	16	7
	**Hand speed peaks bias**	50	50	27	16	7
	**Hand path length bias**	50	50	27	16	7
**BOB task – Level 2**	**Mean bar tilt**	***48***	***48***	***26***	16	7
	**Bar tilt standard deviation**	***48***	***48***	***26***	16	7
	**Bar length variability**	***48***	***48***	***26***	16	7
	**Reaction time difference**	***48***	***48***	***26***	16	7
	**Hand speed difference**	***48***	***48***	***26***	16	7
	**Hand speed peaks bias**	***48***	***48***	***26***	16	7
	**Hand path length bias**	***48***	***48***	***26***	16	7
**OH task**	**Hand transition**	50	50	27	16	7
	**Hand selection overlap**	50	50	27	16	7
	**Hand bias hits**	50	50	27	16	7
	**Hand speed bias**	**49**	**49**	27	16	7
	**Movement area bias**	50	50	27	16	7
**Circuit task**	**Bimanual coordination factor**	***46***	***46***	***26***	15	***5***

BOB task = Ball-on-bar task, OH task = Object hit task, uCP = unilateral cerebral palsy, TDC = typically developing children, MACS = manual Ability Classification System, n = amount of children, bold italic = parameters with missing data
