# Supplementary material for: In-depth quantification of bimanual coordination using the Kinarm exoskeleton robot in children with unilateral cerebral palsy
Source: J Neuroeng Rehabil. 2023 Nov 11;20:154. doi: 10.1186/s12984-023-01278-6 (PMC10640737; doi:10.1186/s12984-023-01278-6)
Supplement: Supplementary file 3 — Additional file 3. Effect sizes of the differences in bimanual coordination between different MACS-levels for all bimanual parameters. Effect sizes (partial eta squared) with their 90% confidence interval from the difference in bimanual coordination in children with uCP with different MACS-levels. For the ANCOVA (A,B): the effect sizes for the main effect of MACS-levels (A) and age (B) is presented. Effect sizes are shown for the moderated regression (C-E) for the interaction between MACS level and age (C), main effect of MACS-level (D) and age (E). Partial eta squared is classified and presented in red (low). yellow (medium) and green (large). A filled in symbol (diamond, circle or square) represent a significant difference with a p ≤ 0.05. MACS = manual ability classification system. [file 12984_2023_1278_MOESM3_ESM.pdf]

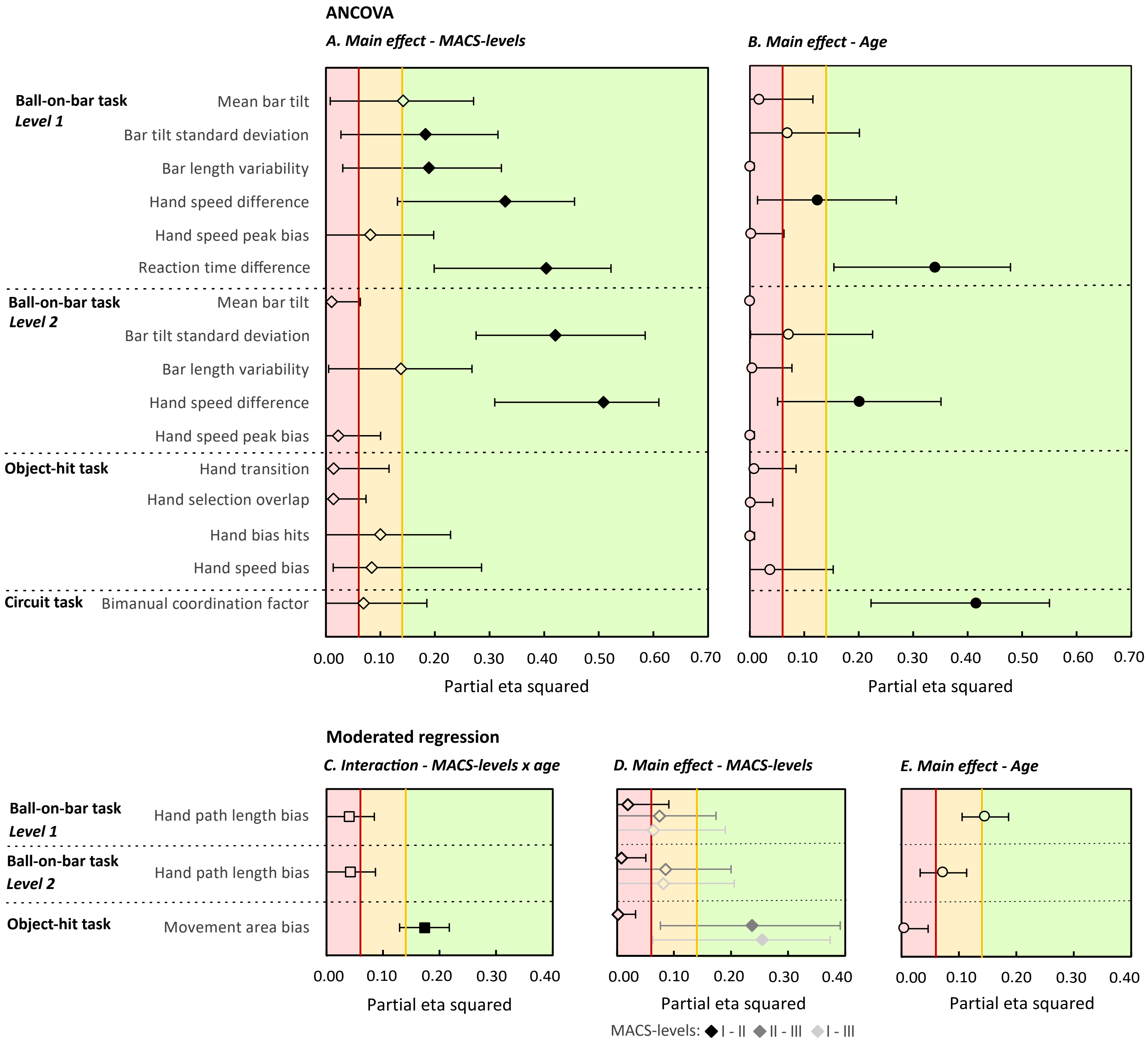

ANCOVA
A. Main effect - MACS-levels
B. Main effect - Age
Ball-on-bar task
Level 1
Mean bar tilt
Bar tilt standard deviation
Bar length variability
Hand speed difference
Hand speed peak bias
Reaction time difference
Ball-on-bar task
Level 2
Mean bar tilt
Bar tilt standard deviation
Bar length variability
Hand speed difference
Hand speed peak bias
Object-hit task
Hand transition
Hand selection overlap
Hand bias hits
Hand speed bias
Circuit task
Bimanual coordination factor
0.00 0.10 0.20 0.30 0.40 0.50 0.60 0.70
Partial eta squared
0.00 0.10 0.20 0.30 0.40 0.50 0.60 0.70
Partial eta squared
Moderated regression
C. Interaction - MACS-levels x age
D. Main effect - MACS-levels
E. Main effect - Age
Ball-on-bar task
Level 1
Hand path length bias
Ball-on-bar task
Level 2
Hand path length bias
Object-hit task
Movement area bias
0.00 0.10 0.20 0.30 0.40
Partial eta squared
0.00 0.10 0.20 0.30 0.40
Partial eta squared
0.00 0.10 0.20 0.30 0.40
Partial eta squared
MACS-levels: I - II II - III I - III
