# Supplementary material for: In-depth quantification of bimanual coordination using the Kinarm exoskeleton robot in children with unilateral cerebral palsy
Source: J Neuroeng Rehabil. 2023 Nov 11;20:154. doi: 10.1186/s12984-023-01278-6 (PMC10640737; doi:10.1186/s12984-023-01278-6)
Supplement: Supplementary file 4 — Additional file 4. Overview of ANCOVA of bimanual parameters between MACS-levels in children with uCP. A full overview of the results of the analysis of covariance of the bimanual parameters in children with uCP with different manual ability classification levels (MACS-levels). uCP = unilateral cerebral palsy, SD = standard deviation, MACS = manual ability level, P-value* = adjusted P-value with false discovery rate of 0.05 for multiple comparison, η2p = partial eta square, bold = significance p ≤ 0.05. [file 12984_2023_1278_MOESM4_ESM.pdf]

Additional file 4: Overview of ANCOVA of bimanual parameters between MACS levels in children with uCP

Tasks		Bimanual parameters	Mean (CI)			MACS					Age	
			Level 1	Level 2	Level 3	F	P-value*<br>(η^2_p)	I vs. II	I vs. III	II vs. III	F	P-value*<br>(η^2_p)
Ball-on-bar task	Level 1	**Mean bar tilt**	0.04<br>(0.02-0.06)	0.06<br>(0.03-0.12)	0.18<br>(0.07-0.50)	3.80	0.06<br>(0.14)				0.79	0.51<br>(0.02)
		**Bar tilt standard deviation**	0.10<br>(0.08-0.13)	0.14<br>(0.10-0.19)	0.24<br>(0.15-0.41)	5.15	**0.02<br>(0.18)**	0.30	**0.01**	0.21	3.41	0.13<br>(0.07)
		**Bar length variability**	3.32<br>(2.84-3.85)	4.17<br>(3.48-4.93)	5.31<br>(4.12-6.66)	5.35	**0.02<br>(0.19)**	0.17	**0.01**	0.34	0.01	0.96<br>(0.00)
		**Hand speed difference**	42.85<br>(39.81-46.13)	51.40<br>(46.77-56.49)	61.66<br>(53.33-71.29)	11.26	**<0.001<br>(0.33)**	**0.01**	**<0.001**	0.12	6.54	0.03<br>(0.12)
		**Hand speed peak bias**	0.03<br>(0.00-0.05)	0.07<br>(0.03-0.10)	0.06<br>(0.01-0.11)	2.06	0.23<br>(0.08)				0.10	0.87<br>(0.00)
		**Reaction time difference**	0.05<br>(0.04-0.06)	0.09<br>(0.07-0.10)	0.11<br>(0.08-0.15)	15.26	**<0.001<br>(0.40)**	**<0.001**	**<0.001**	0.62	23.22	**<0.001<br>(0.34)**
	Level 2	**Mean bar tilt**	0.03<br>(0.02-0.05)	0.04<br>(0.02-0.06)	0.03<br>(0.01-0.06)	0.24	0.87<br>(0.01)				0.00	0.96<br>(0.00)
		Bar tilt standard deviation	0.06<br>(0.06-0.07)	0.08<br>(0.07-0.09)	0.12<br>(0.09-0.16)	20.62	**<0.001<br>(0.36)**	**0.03**	**<0.001**	**0.003**	4.26	0.07<br>(0.09)
		**Bar length variability**	2.88<br>(2.40-3.44)	3.73<br>(2.98-4.68)	4.71<br>(3.31-6.70)	3.60	0.07<br>(0.14)				0.19	0.81<br>(0.00)
		**Hand speed difference**	41.90<br>(38.53-45.28)	54.24<br>(50.00-58.47)	65.46<br>(58.84-72.07)	23.30	**<0.001<br>(0.51)**	**<0.001**	**<0.001**	**0.02**	11.33	**<0.001<br>(0.20)**
		**Hand speed peak bias**	0.02<br>(-0.01-0.05)	0.04<br>(0.01-0.07)	0.02<br>(-0.04-0.07)	0.53	0.74<br>(0.02)				0.01	0.97<br>(0.00)
Object hit task		***Hand transition***	-0.04<br>(-0.05-(-0.02))	-0.03<br>(-0.05-0.00)	-0.02<br>(-0.06-0.02)	0.73	0.83<br>(0.01)				0.05	0.90<br>(0.00)
		**Hand selection overlap**	0.12<br>(0.10-0.14)	0.13<br>(0.10-0.14)	0.12<br>(0.10-0.14)	*0.32*	0.85<br>(0.01)				*1.75*	0.30<br>(0.04)
		**Hand bias hits**	0.15<br>(0.11-0.21)	0.20<br>(0.11-0.21)	0.31<br>(0.11-0.21)	2.57	0.15<br>(0.10)				0.37	0.70<br>(0.01)
		Hand speed bias	0.14<br>(0.09-0.19)	0.22<br>(0.09-0.19)	0.31<br>(0.09-0.19)	4.21	0.20<br>(0.08)				0.01	0.95<br>(0.00)
Circuit task		**Bimanual coordination factor**	0.27<br>(0.26-0.29)	0.26<br>(0.24-0.28)	0.25<br>(0.21-0.28)	1.58	0.32<br>(0.07)				30.54	**<0.001<br>(0.42)**

uCP = unilateral cerebral palsy, SD = standard deviation, MACS = manual ability level, P-value = adjusted P-value with false discovery rate of 0.05 for multiple comparison, η^2_p = partial eta square, bold = significance ≤0.05*
