# Supplementary material for: In-depth quantification of bimanual coordination using the Kinarm exoskeleton robot in children with unilateral cerebral palsy
Source: J Neuroeng Rehabil. 2023 Nov 11;20:154. doi: 10.1186/s12984-023-01278-6 (PMC10640737; doi:10.1186/s12984-023-01278-6)
Supplement: Supplementary file 5 — Additional file 5. Overview of moderated regression of bimanual parameters between MACS_levels in children with uCP. A full overview of the results of the moderated regression of the bimanual parameters in children with uCP with different manual ability classification levels (MACS-levels). MACS = manual ability level, uCP = unilateral cerebral palsy, BOB = Ball-on-bar task, OH = object-hit task, p = p-value with significance ≤ 0.05, R2 = effect size, B = coefficient, t = t-value, bold = significance p ≤ 0.05. [file 12984_2023_1278_MOESM5_ESM.pdf]

Additional file 5: Overview of moderated regression of bimanual parameters between MACS_levels in children with uCP

Bimanual parameters	Model summary	Interaction							MACS-levels						Age	
		Main effect	X1 * age		X2 * age		X3 * age		X1 (I vs. II)		X2 (I vs. III)		X3 (II vs. III)			
	p (R^2)	P (R^2 change)	B (t)	p	B (t)	p	B (t)	p	B (t)	p	B (t)	p	B (t)	p	B (t)	p
BOB – Level 1: Hand path length bias	0.05 (0.26)	0.37 (0.15)	-0.001 (-0.17)	0.87	0.05 (1.40)	0.17	0.05 (1.41)	0.16	0.07 (0.68)	0.50	-0.64 (-1.26)	0.21	-0.71 (-1.38)	0.18	-0.01 (-2.01)	0.05
BOB – Level 2: Hand path length bias	0.27 (0.27)	0.37 (0.23)	0.00 (-0.04)	0.97	0.06 (1.43)	0.16	0.06 (1.43)	0.16	0.02 (0.42)	0.67	-0.78 (-1.43)	0.16	-0.81 (-1.47)	0.15	-0.003 (-1.34)	0.19
**OH: Movement area bias**	**0.02** (0.27)	**0.01** (0.16)	-0.002 (-0.35)	0.73	-0.04 (-3.13)	**0.003**	-0.04 (-2.94)	**0.01**	0.02 (0.22)	0.82	0.53 (2.84)	**0.01**	0.51 (2.70)	**0.01**	0.001 (0.38)	0.71

MACS = manual ability level, uCP = unilateral cerebral palsy, BOB = Ball-on-bar task, OH = object-hit task, p = p-value, R^2 = effect size, B = coefficient, t = t-value, bold = significance ≤0.05
