# Supplementary material for: In-depth quantification of bimanual coordination using the Kinarm exoskeleton robot in children with unilateral cerebral palsy
Source: J Neuroeng Rehabil. 2023 Nov 11;20:154. doi: 10.1186/s12984-023-01278-6 (PMC10640737; doi:10.1186/s12984-023-01278-6)
Supplement: Supplementary file 6 — Additional file 6. Correlation coefficients of all unimanual and task execution parameters with the bimanual parameters. Pearson and spearman's rank correlation coefficients of the unimanual and task execution parameters with the bimanual parameters of the ball-on-bar task (A), object-hit task (B) and Circuit task (C) for the selected parameters in children with uCP. DH = dominant hand, NDH = non-dominant hand, s = spearman’s rank correlation. [file 12984_2023_1278_MOESM6_ESM.pdf]

A. Ball-on-bar task

Level	Variable	Targets complete (Execution)	Drops (Execution)	Time to target (Execution)	Ball speed (Execution)	Hand speed (Unimanual DH)	Hand speed peaks (Unimanual DH)	Hand speed (Unimanual NDH)	Hand speed peaks (Unimanual NDH)
Level 1	Mean bar tilt	**-0.31**	-	**0.33**	-0.11 S	0.12 S	0.26 S	-0.17 S	0.16 S
	Bar tilt standard deviation	***-0.63*** S	-	***0.54*** S	-0.17 S	0.13 S	0.05 S	-0.21 S	-0.01 S
	Bar length variability	-0.15 S	-	0.14 S	0.18 S	**0.33** S	***0.43*** S	0.18 S	0.13 S
	Hand speed difference	***-0.46*** S		***0.41*** S	0.00 S	**0.33** S	0.17 S	-0.07 S	0.11 S
	Hand speed peaks bias	-0.06 S	-	0.02 S	0.25 S	0.13 S	***0.38*** S	**0.33** S	***-0.47*** S
	Hand path length bias	***-0.47***	-	**0.54**	**-0.34**	0.18	-0.05	***-0.60***	0.18
	Reaction time difference	***-0.77*** S	-	***0.72*** S	***-0.45*** S	-0.23 S	-0.11 S	***-0.44*** S	**-0.33** S
Level 2	Mean bar tilt	-0.21 S	0.15	0.13 S	-0.08 S	-0.06 S	-0.04 S	-0.04 S	0.08 S
	Bar tilt standard deviation	***-0.54*** S	***0.61***	***0.46*** S	0.11 S	-0.03 S	0.03 S	-0.07 S	0.01 S
	Bar length variability	-0.12 S	***0.44***	0.13 S	**0.28** S	0.26 S	***0.40*** S	0.27 S	0.22 S
	Hand speed difference	**-0.63** S	***0.38***	**0.58** S	-0.11 S	-0.23 S	0.11 S	-0.26 S	0.16 S
	Hand speed peaks bias	0.17 S	-0.18	-0.13 S	-0.05	0.03 S	***0.48*** S	0.12 S	-0.23 S
	Hand path length bias	***-0.40*** S	0.10	**0.37** S	-0.15 S	-0.01 S	-0.23 S	***-0.48*** S	0.16 S

B. Object-hit task

Variable	Target hits (Execution)	Median error (Execution)	Miss bias (Execution)	Hits (Unimanual DH)	Hand speed (Unimanual DH)	Movement area (Unimanual DH)	Hits (Unimanual NDH)	Hand speed (Unimanual NDH)	Movement area (Unimanual NDH)
Hand bias hits	**-0.44**	**-0.35**	0.06 S	-0.09	***0.41*** S	***0.47*** S	***-0.75***	***-0.37*** S	-0.20 S
Hand transition	0.20	0.29	***-0.62*** S	0.05	***-0.43*** S	***-0.59*** S	**0.32**	-0.02 S	0.13 S
Movement area bias	-0.24	-0.20	***0.40*** S	-0.02	0.27 S	***0.42*** S	***-0.44***	***-0.47*** S	***-0.62*** S
Hand speed bias	***-0.46***	**-0.31**	0.18 S	-0.19	0.28 S	**0.34** S	***-0.66***	***-0.65*** S	***-0.50*** S
Hand selection overlap	-0.27	-0.16	-0.02 S	-0.24	**0.32** S	***0.41*** S	-0.25	***0.43*** S	***0.49*** S

B. Circuit task

Variable	Error	Velocity	Skill
Bimanual coordination factor	0,24	0,03 S	-0,19 S

Correlation Coefficient

1,00
0,90
0,70
0,50
0,30
0,00
-0,30
-0,50
-0,70
-0,90
-1,00
